# Spontaneous coronary artery dissection of ramus intermedius in an elderly woman^☆^

**DOI:** 10.1016/j.radcr.2022.07.092

**Published:** 2022-09-06

**Authors:** Carl Tanba, Sumanth Bandaru, Zayd Alhaddad, Fady Iskander

**Affiliations:** a Department of Internal Medicine, Medstar Union Memorial Hospital, 301 E University Pkwy, Baltimore, MD 21218, USA; b Department of Cardiology, Medstar Union Memorial Hospital, 3333 North Calvert Street Johnston Professional Bldg Ste LL08, Baltimore, MD, 21218, USA

**Keywords:** Spontaneous coronary artery dissection, Coronary angiography, Chest pain, Acute coronary syndrome

## Abstract

Spontaneous coronary artery dissection (SCAD) is a rare cause of myocardial ischemia commonly seen in younger patients, particularly women. Patients often present similar to those with acute coronary syndrome (ACS); however, they often are missing the classic risk factors that are typically associated with coronary artery disease. Differentiating between SCAD and ACS is vital as they are managed differently with up to 80% of SCAD being managed conservatively. We present a case of 61-year-old woman with no previous cardiac history presenting with chest pain and was found to have spontaneous coronary artery dissection on coronary angiography.

## Introduction

Spontaneous coronary artery dissection (SCAD) is a rare cause of myocardial ischemia. While it usually presents similar to acute coronary syndrome, it occurs in patients without any traditional risk factors for atherosclerosis. SCAD is predominantly seen in female patients especially in peripartum women in their 40s or 50s [1]. The exact pathophysiology is not understood. Most patients have a history of hypertension and present with elevated blood pressure in the acute event. SCAD has a high mortality risk at 1%. Diagnosis is made through coronary angiography which can differentiate between plaque rupture and coronary artery dissection. Further management involves decreasing the risk of recurrence but there is no consensus on an overall approach [2].

## Case report

Sixty-one-year-old woman with no prior cardiac history presented to the emergency department with chest pain. One day prior, she had intermittent chest pain 5/10 in intensity nonradiating which improved with antacids. On the day of presentation, she woke up with sudden onset severe left-sided chest pain 8 out of 10 in intensity, radiating to the left shoulder associated with shortness of breath and palpitations. She tried taking antacids but her symptoms persisted so she decided to seek medical care.

She has been having intermittent chest pain for the past 2 years which she attributed to gastroesophageal reflux disease and was taking antacids with some relief. She did not seek any medical attention for her chest pain prior to this episode. She is an active cigarette smoker with more than 40 pack years and occasionally smokes marijuana, denies alcohol use. She works as a physical therapist and has no family history of cardiac disease or sudden cardiac death.

On arrival, her heart rate was 96 bpm, she was markedly hypertensive with a blood pressure of 170/100 mmHg and was saturating 97% on room air. She was given 325 mg of aspirin and 2 tablets 0.4 mg of sublingual nitroglycerin with minimal relief. Due to persistent chest pain, nitroglycerin drip was started.

**Fig. 1:**
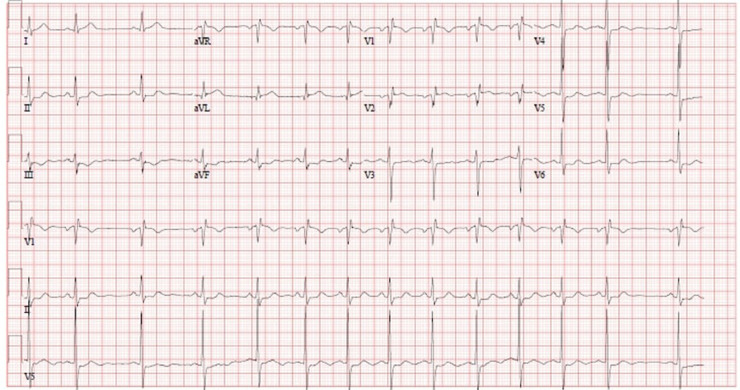
Twelve-lead electrocardiogram (EKG) showed 2 mm ST depression in lead II, III, and aVF along with right bundle branch block.

Electrocardiogram (EKG) (Fig. 1) showed 2 mm ST depression in lead II, III, and aVF along with incomplete right bundle branch block with no previous EKGs for comparison.

Complete blood count was significant for leukocytosis of 12.5 k/uL with 80.5% neutrophils and basic metabolic panel was unremarkable. Initial high sensitivity troponin-I was 936 ng/L. COVID-19 test along with Influenza A were negative.

Chest X-ray did not show any acute intrapulmonary process. A diagnosis of non-ST elevation myocardial infarction was made, she was admitted to the intensive care unit for close monitoring and was started on heparin drip, metoprolol tartrate 25 mg.

She continued to have persistent chest pain despite nitroglycerin drip and her troponin continued to rise; therefore, she was urgently transferred to our facility for emergency cardiac catheterization.

She underwent cardiac catheterization which showed severe single vessel coronary artery disease involving 99% narrowing of the ramus intermedius with appearance suggestive of SCAD (Fig. 2 and Video 1), given small caliber vessel and long diffuse narrowing. Cardiac catheterization also showed left ventricle end diastolic pressure of 36 mmHg; therefore, she received a single dose of 40 mg IV Lasix. Postcatheterization EKG revealed normal sinus rhythm with resolution of ST segment depressions.

**Fig, 2:**
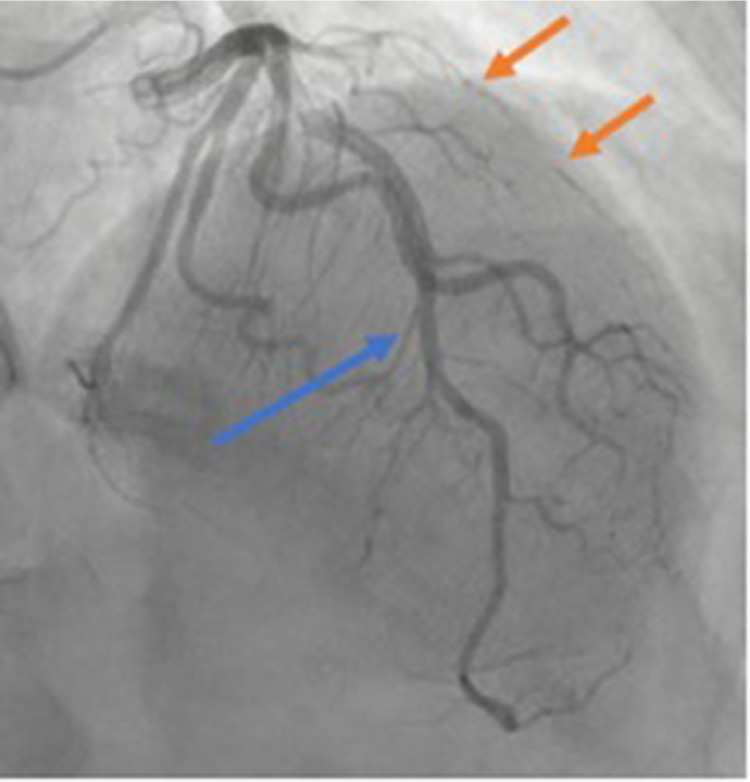
Coronary angiography showed severe single vessel coronary artery disease involving 99% narrowing of the ramus intermedius (orange arrow) with nonobstructive disease of the left coronary distribution including the left anterior descending (LAD).

Troponin eventually peaked at 38,766 ng/l, she was medically managed with aspirin, plavix, statin and beta blocker along with 48 hours of heparin drip since the dissected vessel was not suitable for percutaneous coronary intervention. Transthoracic echocardiogram done prior to discharge showed mild left ventricular hypertrophy with ejection fraction of 65%-70% with no obvious wall motion abnormalities. She was discharged 3 days after hospitalization and followed up with the cardiologist a month later. At the time of follow-up, she had no symptoms and EKG showed normal sinus rhythm with incomplete right bundle branch pattern.

## Discussion

Blood supply to the heart is by coronary arteries including the right coronary and left coronary artery. The left main coronary artery arises from left coronary sinus and bifurcates into left anterior descending (LAD) & left circumflex arteries (LCX). In about 15%-30% of patients left main coronary artery trifurcates into LAD, LCX & ramus intermedius artery as seen in our patient [3,4].

SCAD is defined as nontraumatic, noniatrogenic, spontaneous splitting of the coronary artery wall. It leads to formation of false lumen filled with intramural hematoma leading to narrowing of the artery and diminishing the blood flow [5].

The first case of SCAD was described by Pretty in 1931 [6]. Since then several cases have been reported but it is still considered to be a rare phenomenon. As per one review 90% of patients with SCAD are women aged between 47 and 53 years of age. Risk factors for SCAD include connective tissue disease, inflammatory bowel disease, celiac disease, pregnancy, & postpartum [7]. Patients with SCAD can present to the emergency department with acute coronary syndrome, ventricular fibrillation, cardiogenic shock and even sudden cardiac death [1,7]. High index of suspicion for diagnosis is necessary as patients with SCAD are usually young with no atherosclerotic risk factors, they can often be misdiagnosed and it can lead to fatal outcomes.

Cardiac catheterization with coronary angiogram is the initial diagnostic test. SCAD is classified into 3 types based on angiographic findings by Saw. Type 1 demonstrates multiple radiolucent lumens; type 2 shows diffuse stenosis of the vessel and type 3 often mimics atherosclerosis and can be misdiagnosed [8]. Intracoronary diagnostic testing such as optical coherence tomography and intravascular ultrasound directly visualize the arterial wall and significantly improve the diagnostic accuracy. They are especially useful in diagnosing types 2 and 3 SCAD as these can cause diagnostic uncertainty [8].

Most patients with SCAD are managed conservatively without percutaneous coronary intervention (PCI), as there is evidence of complete spontaneously healing of the affected arteries. [9]. PCI is done in special situations such as complete vessel occlusion, hemodynamic instability, and recurrent ischemia [10].

## Patient consent

I state that written and informed consent was taken from the patient for publication of this case. The patient was informed that no personal details will be revealed in the publishing of this case.

## Appendix Supplementary materials

Image, application 1
